# Early role of vascular dysregulation on late-onset Alzheimer's disease based on multifactorial data-driven analysis

**DOI:** 10.1038/ncomms11934

**Published:** 2016-06-21

**Authors:** Y. Iturria-Medina, R. C. Sotero, P. J. Toussaint, J. M. Mateos-Pérez, A. C. Evans, The Alzheimer's Disease Neuroimaging Initiative, Michael W. Weiner, Paul Aisen, Ronald Petersen, Clifford R. Jack, William Jagust, John Q. Trojanowki, Arthur W. Toga, Laurel Beckett, Robert C. Green, Andrew J. Saykin, John Morris, Leslie M. Shaw, Zaven Khachaturian, Greg Sorensen, Lew Kuller, Marc Raichle, Steven Paul, Peter Davies, Howard Fillit, Franz Hefti, Davie Holtzman, M Marcel Mesulam, William Potter, Peter Snyder, Adam Schwartz, Tom Montine, Ronald G. Thomas, Michael Donohue, Sarah Walter, Devon Gessert, Tamie Sather, Gus Jiminez, Danielle Harvey, Matthew Bernstein, Nick Fox, Paul Thompson, Norbert Schuff, Bret Borowski, Jeff Gunter, Matt Senjem, Prashanthi Vemuri, David Jones, Kejal Kantarci, Chad Ward, Robert A. Koeppe, Norm Foster, Eric M. Reiman, Kewei Chen, Chet Mathis, Susan Landau, Nigel J. Cairns, Erin Householder, Lisa Taylor-Reinwald, Virginia Lee, Magdalena Korecka, Michal Figurski, Karen Crawford, Scott Neu, Tatiana M. Foroud, Steven Potkin, Li Shen, Kelley Faber, Sungeun Kim, Kwangsik Nho, Leon Thal, Neil Buckholtz, Marylyn Albert, Richard Frank, John Hsiao, Jeffrey Kaye, Joseph Quinn, Betty Lind, Raina Carter, Sara Dolen, Lon S. Schneider, Sonia Pawluczyk, Mauricio Beccera, Liberty Teodoro, Bryan M. Spann, James Brewer, Helen Vanderswag, Adam Fleisher, Judith L. Heidebrink, Joanne L. Lord, Sara S. Mason, Colleen S. Albers, David Knopman, Kris Johnson, Rachelle S. Doody, Javier Villanueva-Meyer, Munir Chowdhury, Susan Rountree, Mimi Dang, Yaakov Stern, Lawrence S. Honig, Karen L. Bell, Beau Ances, Maria Carroll, Sue Leon, Mark A. Mintun, Stacy Schneider, Angela Oliver, Daniel Marson, Randall Griffith, David Clark, David Geldmacher, John Brockington, Erik Roberson, Hillel Grossman, Effie Mitsis, Leyla de Toledo-Morrell, Raj C. Shah, Ranjan Duara, Daniel Varon, Maria T. Greig, Peggy Roberts, Marilyn Albert, Chiadi Onyike, Daniel D'Agostino, Stephanie Kielb, James E. Galvin, Brittany Cerbone, Christina A. Michel, Henry Rusinek, Mony J. de Leon, Lidia Glodzik, Susan De Santi, P. Murali Doraiswamy, Jeffrey R. Petrella, Terence Z. Wong, Steven E. Arnold, Jason H. Karlawish, David Wolk, Charles D. Smith, Greg Jicha, Peter Hardy, Partha Sinha, Elizabeth Oates, Gary Conrad, Oscar L. Lopez, MaryAnn Oakley, Donna M. Simpson, Anton P. Porsteinsson, Bonnie S. Goldstein, Kim Martin, Kelly M. Makino, M. Saleem Ismail, Connie Brand, Ruth A. Mulnard, Gaby Thai, Catherine Mc-Adams-Ortiz, Kyle Womack, Dana Mathews, Mary Quiceno, Ramon Diaz-Arrastia, Richard King, Myron Weiner, Kristen Martin-Cook, Michael DeVous, Allan I. Levey, James J. Lah, Janet S. Cellar, Jeffrey M. Burns, Heather S. Anderson, Russell H. Swerdlow, Liana Apostolova, Kathleen Tingus, Ellen Woo, Daniel H. S. Silverman, Po H. Lu, George Bartzokis, Neill R. Graff-Radford, Francine Parfitt, Tracy Kendall, Heather Johnson, Martin R. Farlow, AnnMarie Hake, Brandy R. Matthews, Scott Herring, Cynthia Hunt, Christopher H. van Dyck, Richard E. Carson, Martha G. MacAvoy, Howard Chertkow, Howard Bergman, Chris Hosein, Sandra Black, Bojana Stefanovic, Curtis Caldwell, Ging-Yuek Robin Hsiung, Howard Feldman, Benita Mudge, Michele Assaly, Andrew Kertesz, John Rogers, Charles Bernick, Donna Munic, Diana Kerwin, Marek-Marsel Mesulam, Kristine Lipowski, Chuang-Kuo Wu, Nancy Johnson, Carl Sadowsky, Walter Martinez, Teresa Villena, Raymond Scott Turner, Kathleen Johnson, Brigid Reynolds, Reisa A. Sperling, Keith A. Johnson, Gad Marshall, Meghan Frey, Barton Lane, Allyson Rosen, Jared Tinklenberg, Marwan N. Sabbagh, Christine M. Belden, Sandra A. Jacobson, Sherye A. Sirrel, Neil Kowall, Ronald Killiany, Andrew E. Budson, Alexander Norbash, Patricia Lynn Johnson, Joanne Allard, Alan Lerner, Paula Ogrocki, Leon Hudson, Evan Fletcher, Owen Carmichael, John Olichney, Charles DeCarli, Smita Kittur, Michael Borrie, T-Y Lee, Rob Bartha, Sterling Johnson, Sanjay Asthana, Cynthia M. Carlsson, Steven G. Potkin, Adrian Preda, Dana Nguyen, Pierre Tariot, Stephanie Reeder, Vernice Bates, Horacio Capote, Michelle Rainka, Douglas W. Scharre, Maria Kataki, Anahita Adeli, Earl A. Zimmerman, Dzintra Celmins, Alice D. Brown, Godfrey D. Pearlson, Karen Blank, Karen Anderson, Robert B. Santulli, Tamar J. Kitzmiller, Eben S. Schwartz, Kaycee M. Sink, Jeff D. Williamson, Pradeep Garg, Franklin Watkins, Brian R. Ott, Henry Querfurth, Geoffrey Tremont, Stephen Salloway, Paul Malloy, Stephen Correia, Howard J. Rosen, Bruce L. Miller, Jacobo Mintzer, Kenneth Spicer, David Bachman, Elizabether Finger, Stephen Pasternak, Irina Rachinsky, Dick Drost, Nunzio Pomara, Raymundo Hernando, Antero Sarrael, Susan K. Schultz, Laura L. Boles Ponto, Hyungsub Shim, Karen Elizabeth Smith, Norman Relkin, Gloria Chaing, Lisa Raudin, Amanda Smith, Kristin Fargher, Balebail Ashok Raj, Thomas Neylan, Jordan Grafman, Melissa Davis, Rosemary Morrison, Jacqueline Hayes, Shannon Finley, Karl Friedl, Debra Fleischman, Konstantinos Arfanakis, Olga James, Dino Massoglia, J. Jay Fruehling, Sandra Harding, Elaine R. Peskind, Eric C. Petrie, Gail Li, Jerome A. Yesavage, Joy L. Taylor, Ansgar J. Furst

**Affiliations:** 1 Department of Neurology & Neurosurgery, McConnell Brain Imaging Centre, Montreal Neurological Institute, Montreal, Quebec, Canada H3A 2B4; 2 Ludmer Centre for NeuroInformatics and Mental Health, Montreal, Quebec, Canada H3A 2B4; 3 Department of Radiology and Hotchkiss Brain institute, University of Calgary, Calgary, Alberta, Canada T2N 4N1; 4 UC San Francisco, California, USA; 5 UC San Diego, California, USA; 6 Mayo Clinic, Rochester, New York, USA; 7 UC Berkeley, California, USA; 8 UPenn, Philadelphia, Pennsylvania, USA; 9 USC, Los Angeles, California, USA; 10 UC Davis, California, USA; 11 Brigham and Women's Hospital/Harvard Medical School, Boston, Massachusetts, USA; 12 Indiana University, Bloomington, Indiana, USA; 13 Washington University St Louis, Missouri, USA; 14 Prevent Alzheimer's Disease 2020, Rockville, Maryland, USA; 15 Siemens, Munich, Germany; 16 University of Pittsburg, Pennsylvania, USA; 17 Cornell University, Ithaca, New York, USA; 18 Albert Einstein College of Medicine of Yeshiva University, Bronx, New York, USA; 19 AD Drug Discovery Foundation, New York City, New York, USA; 20 Acumen Pharmaceuticals, Livermore, California, USA; 21 Northwestern University, Evanston and Chicago, Illinois, USA; 22 National Institute of Mental Health, Rockville, Maryland, USA; 23 Brown University, Providence, Rhode Island, USA; 24 Eli Lilly, Indianapolis, Indiana, USA; 25 University of Washington, Seattle, Washington, USA; 26 University of London, London, England; 27 UCLA, Los Angeles, California, USA; 28 University of Michigan, Ann Arbor, Michigan, USA; 29 University of Utah, Salt Lake, Utah, USA; 30 Banner Alzheimer's Institute, Phoenix, Arizona, USA; 31 UC Irvine, Irvine, California, USA; 32 National Institute on Aging, Bethesda, Maryland, USA; 33 Johns Hopkins University, Baltimore, Maryland, USA; 34 Richard Frank Consulting, Washington, DC, USA; 35 Oregon Health and Science University, Portland, Oregon, USA; 36 Baylor College of Medicine, Houston, Texas, USA; 37 University of Alabama, Birmingham, Alabama, USA; 38 Mount Sinai School of Medicine, New York City, New York, USA; 39 Rush University Medical Center, Chicago, Illinois, USA; 40 Wien Center, Miami, Florida, USA; 41 New York University, New York City, New York, USA; 42 Duke University Medical Center, Durham, North Carolina, USA; 43 University of Kentucky, Lexington, Kentucky, USA; 44 University of Rochester Medical Center, Rochester, New York, USA; 45 University of Texas Southwestern Medical School, Dallas, Texas, USA; 46 Emory University, Atlanta, Georgia, USA; 47 University of Kansas, Medical Center, Kansas City, Kansas, USA; 48 Mayo Clinic, Jacksonville, Florida, USA; 49 Yale University School of Medicine, New Haven, Connecticut, USA; 50 McGill University/Montreal-Jewish General Hospital, Montreal, Quebec, Canada; 51 Sunnybrook Health Sciences, Toronto, Ontario, Canada; 52 U.B.C. Clinic for AD & Related Disorders, Vancouver, British Columbia, Canada; 53 Cognitive Neurology–St Joseph's, London, Ontario, Canada; 54 Cleveland Clinic Lou Ruvo Center for Brain Health, Las Vegas, Nevada, USA; 55 St Joseph's Health Care, London, Ontario, Canada; 56 Premiere Research Institute, Palm Beach Neurology, Miami, Florida, USA; 57 Georgetown University Medical Center, Washington, DC, USA; 58 Banner Sun Health Research Institute, Sun City, Arizona, USA; 59 Boston University, Boston, Massachusetts, USA; 60 Howard University, Washington, DC, USA; 61 Case Western Reserve University, Cleveland, Ohio, USA; 62 Neurological Care of CNY, Liverpool, New York, USA; 63 Parkwood Hospital, London, Ontario, USA; 64 University of Wisconsin, Madison, Wisconsin, USA; 65 Dent Neurologic Institute, Amherst, New York, USA; 66 Ohio State University, Columbus, Ohio, USA; 67 Albany Medical College, Albany, New York, USA; 68 Hartford Hospital, Olin Neuropsychiatry Research Center, Hartford, Connecticut, USA; 69 Dartmouth-Hitchcock Medical Center, Lebanon, New Hampshire, USA; 70 Wake Forest University Health Sciences, Winston-Salem, North Carolina, USA; 71 Rhode Island Hospital, Providence, Rhode Island, USA; 72 Butler Hospital, Providence, Rhode Island, USA; 73 Medical University South Carolina, Charleston, South Carolina, USA; 74 Nathan Kline Institute, Orangeburg, New York, USA; 75 University of Iowa College of Medicine, Iowa City, Iowa, USA; 76 University of South Florida: USF Health Byrd Alzheimer's Institute, Tampa, Florida, USA; 77 Department of Defense, Arlington, Virginia, USA; 78 Stanford University, Stanford, California, USA

## Abstract

Multifactorial mechanisms underlying late-onset Alzheimer's disease (LOAD) are poorly characterized from an integrative perspective. Here spatiotemporal alterations in brain amyloid-β deposition, metabolism, vascular, functional activity at rest, structural properties, cognitive integrity and peripheral proteins levels are characterized in relation to LOAD progression. We analyse over 7,700 brain images and tens of plasma and cerebrospinal fluid biomarkers from the Alzheimer's Disease Neuroimaging Initiative (ADNI). Through a multifactorial data-driven analysis, we obtain dynamic LOAD–abnormality indices for all biomarkers, and a tentative temporal ordering of disease progression. Imaging results suggest that intra-brain vascular dysregulation is an early pathological event during disease development. Cognitive decline is noticeable from initial LOAD stages, suggesting early memory deficit associated with the primary disease factors. High abnormality levels are also observed for specific proteins associated with the vascular system's integrity. Although still subjected to the sensitivity of the algorithms and biomarkers employed, our results might contribute to the development of preventive therapeutic interventions.

## Abstract (web summary)

Late-onset Alzheimer's disease (LOAD) is a complex multi-factorial disorder. Here, the authors perform a data-driven analysis of LOAD progression, including multimodal brain imaging, plasma and CSF biomarkers, and find vascular dysfunction is among the earliest and strongest altered events.

## Graphical abstract

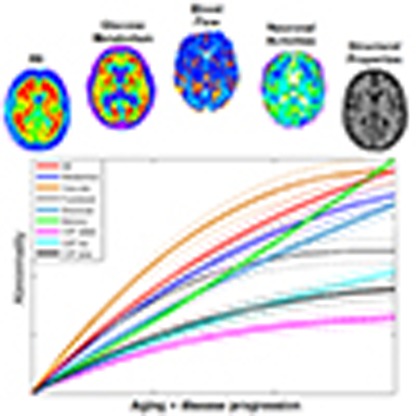

Late-onset Alzheimer's disease (LOAD), the most common form of human dementia, is not causally associated with any unique neuropathological mechanism but rather with multiple concomitant factors. The high complexity of the mechanisms underlying the disease and the current lack of quantitative integrative models comparing them make our understanding of LOAD outcome/progression and the development of effective disease-modifying therapeutic agents difficult.

Historically, different hypotheses for the origin of the disease have been proposed and the most consistent are still the subject of scientific debate^1^,^2^,^3^. The vascular dysregulation hypothesis, dating from the early 1900s, proposes alterations to the balance between the blood flow substrate delivery and the neuronal/glial energy demands, which lead to brain dysfunction and disease^3^,^4^,^5^. Alternatively, amyloid-β (Aβ) and tau misfolded proteins are thought to have a causal role on the cascade of cognitive/clinical events leading to LOAD^2^,^6^. The metabolic dysregulation hypothesis postulates impaired compensatory mechanisms associated with neuronal/glial energy production^7^. More recently, neuronal activity-dependent degeneration mechanisms have been postulated to explain the pathology as a consequence of neuronal/synaptic hyperactivity that expands a ‘toxic' effect on surrounding connected neurons/synapses^1^,^8^. Tissular neurodegeneration and associated grey matter atrophy are other common hallmarks of disease progression, although their causes and roles are not totally understood, being generally thought of as a consequence of previous neuropathological factors.

Despite their importance, models that define a multifactorial LOAD pathogenesis^9^,^10^,^11^,^12^,^13^,^14^ have generally been based on limited data that do not cover the multiplicity of possible biological factors that influence disease progression. For instance, in Jack *et al*.^10^, one of the most cited models of LOAD progression, the vascular dysregulation and the functional impairment components are ignored, even when these factors were historically and consistently associated with the disease's underlying mechanisms^1^,^3^,^4^,^5^,^8^. Motivated by this lack of an integrative LOAD description, here we propose a multifactorial data-driven analysis (MFDDA) approach, wherein alteration levels of Aβ misfolded proteins, metabolism, vascular regulation, functional activity at rest, structural tissue properties and protein levels are spatiotemporally characterized in relation to LOAD progression.

We analysed over 7,700 multimodality brain images and tens of different plasma and cerebrospinal fluid (CSF) biomarkers from 1,171 healthy and diseased subjects. Comparing the characteristic trajectory of each imaging or biospecimen biomarker in pathologic versus healthy aging, our data-driven approach revealed a multifactorial temporal ordering of disease progression. According to this ordering, and under the assumption that the analysed biomarkers represent specific physiological processes, vascular dysregulation might be the earliest/strongest brain pathologic factor associated with LOAD development, followed in order by Aβ deposition, glucose metabolism dysregulation, functional impairment, and grey matter atrophy. Symptoms of cognitive decline were observed from initial LOAD stages, suggesting a continuous memory deterioration caused by subtle pathological alterations in the primary disease factors (for example, vascular/metabolic dysregulation and Aβ effects). Our plasma and CSF results suggest the presence of early peripheral vascular alterations in the disease, and reveal new evidence about inflammatory activation, insulin resistance and associated lipid metabolism dysfunction. Finally, we highlight current limitations and challenges associated with the multifactorial modelling of LOAD. In addition to improving our understanding of LOAD, the methodology proposed in this study could be used for the analysis of other devastating human neurodegenerative disorders.

## Results

### Capturing abnormal biomarker trajectories in unhealthy aging

**Figure 1:**
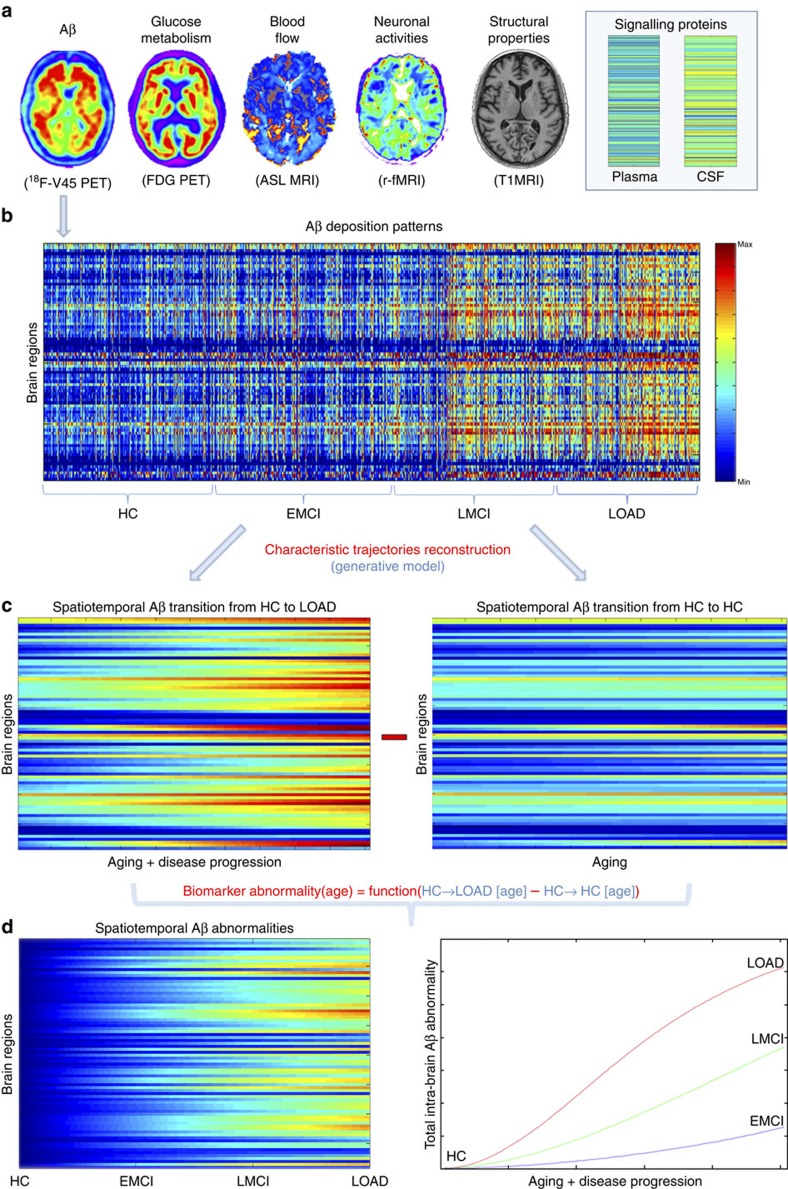
Representation of the multifactorial data-driven generative approach. (**a**) Brain multimodality images and plasma/CSF biomarkers. (**b**) Regional patterns for Aβ deposition across the entire sample. (**c**) Reconstructed regional Aβ characteristic trajectories for HC to LOAD (left) and HC to HC (right) clinical transitions, over a 30-year aging period. (**d**) Regional (left) and total (right) Aβ abnormality trajectories during the age-mediated clinical transitions.

We evaluated Aβ misfolded proteins, glucose metabolism, cerebral blood flow, functional activity and/or structural tissue brain patterns in a cohort of 1,171 subjects from the ADNI database (Methods section, Study participants; Supplementary Table 1). These five biological factors were mapped *in vivo* using corresponding neuroimaging techniques (Fig. 1a; Methods section, Data Description and Processing): Florbetapir positron emission tomography (PET; for Aβ deposition), Fluorodeoxyglucose PET (for glucose metabolism), Arterial Spin Labeling (ASL, for cerebral blood flow), resting functional magnetic resonance imaging (MRI; for neuronal activity at rest) and structural MRI (for structural tissular properties). Each participant was previously diagnosed at each visit as healthy control (HC), early mild cognitive impairment (EMCI), late mild cognitive impairment (LMCI) or probable Alzheimer's disease patient (LOAD). In addition, participants were cognitively and genetically characterized (for example, according to the Mini Mental State Examination (MMSE) or to the number of *apoeɛ4* allele copies, respectively). See Supplementary Table 1 for a detailed sample description across all data modalities. For each mentioned biological factor, representative regional values were calculated for 78 regions covering all the grey matter^15^. See Methods section (image processing subsections) for a description of evaluated multimodality imaging measurements.

**Figure 2:**
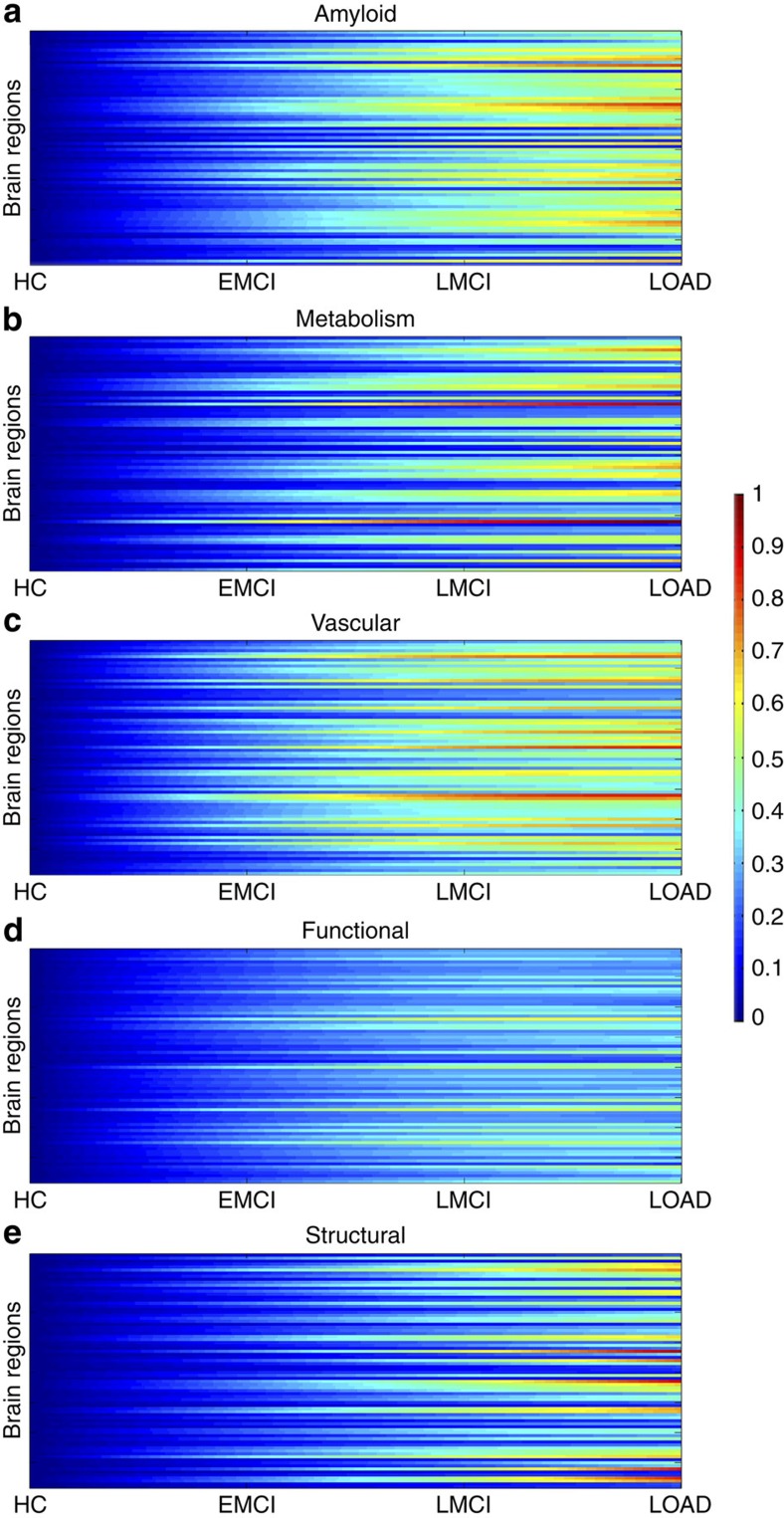
Spatiotemporal abnormalities for LOAD progression (HC to AD clinical transitions) over a 30-year aging period. Regional abnormality trajectories and LOAD–abnormality indices for Aβ deposition (**a**), metabolic dysfunction (**b**), vascular dysregulation (**c**), functional impairment (**d**) and grey matter atrophy (**e**).

We proceeded to reconstruct the characteristic trajectory of each biological factor at each brain region during healthy or unhealthy aging. For this, different aging-mediated disease trajectories were generated using a generative spatiotemporal model (Fig. 1b–d; MFDDA, Methods section and Supplementary Note 1), covering all possible LOAD-associated clinical state transitions during a 30-year period of aging (from 40 to 70 years of age). Clinical transitions considered were: HC to HC, HC to EMCI, HC to LMCI and HC to LOAD state (Fig. 1c). Next, spatiotemporal abnormality trajectories for each specific biomarker were obtained comparing the mean characteristic curve for each diseased clinical transition with the corresponding curve for the healthy aging transition (Fig. 2a–e; MFDDA, Methods section). In addition, these trajectories were used to calculate a total LOAD–abnormality index for each biological factor and brain region, that is, the normalized area under the obtained abnormality curve (MFDDA, Methods section).

This generative procedure was repeated 500 times via a bootstrapping technique, which improved the robustness of the estimations and allowed to control the stability of the results. Finally, each factor-regional trajectory and associated abnormality scores were calculated as the mean of all the bootstrap outcomes. Similarly, aging/disease characteristic trajectories and corresponding abnormality trajectories/scores were generated for MMSE, included as a measure of symptoms severity^13^, and for 146 plasma and 87 CSF potential biomarkers (Supplementary Tables 2 and 3). For further details see Fig. 1, Methods section (MFDDA subsection) and Supplementary Note 1.

### Multifactorial biomarker ordering in LOAD progression

Identification of the sequence of pathological events underlying LOAD progression is still a major challenge (Fig. 2). In the last decade, different models have been proposed^9^,^10^,^11^,^12^,^13^,^14^,^16^. These studies have contributed to the understanding of the ordering in biomarker abnormalities associated with LOAD, using different observational^9^,^10^ or data-driven perspectives^11^,^12^,^13^,^14^. In addition to being less sensitive to subjective criteria, data-driven models present the advantage of being directly applicable to different diseases. For example, in refs 11, 12 a probabilistic event-based model is applied to Alzheimer's and Huntington's cohorts, providing disease-specific pathologic event orderings and individual disease states, without the assumption of *a priori* event ordering or requiring an initial grouping of the patients into clinical stages.

However, in general, previous models of LOAD progression considered an insufficient number of interrelated neuropathological factors and/or brain areas. For example, it is common to find multimodal analyses of grey matter atrophy, Aβ deposition and/or functional impairment, in which vascular dysregulation is not included (see refs 1, 9, 10, 16). In other studies^14^,^17^, conclusions have been based only on the observation of specific brain areas, those presumably more affected in advanced LOAD states (for example, hippocampal, ventricles and entorhinal regions). In refs 11, 12, the analyses were limited to structural, cognitive, Aβ classification in negative and positive subjects, and/or a few peripheral protein biomarkers (CSF Aβ_1–42_, tau and phosphorylated tau (ptau)); brain vascular, functional and metabolic components, as well as other relevant peripheral protein biomarkers, were not considered. See the Discussion section for further description of previous models. Motivated by this lack of integrative LOAD models, here we aimed to identify a comprehensive multifactorial biomarker ordering in LOAD progression based on the spatiotemporal abnormality levels obtained previously for the whole HC to LOAD clinical transition.

**Figure 3:**
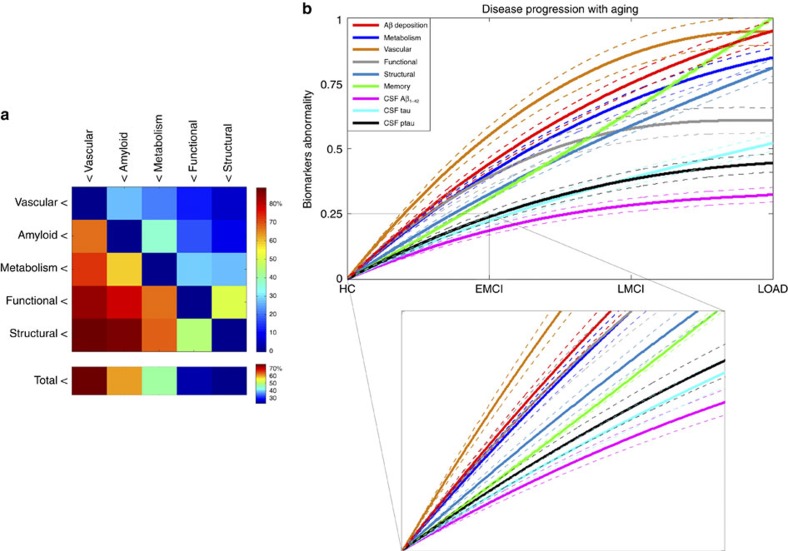
Data-driven spatiotemporal ordering in LOAD progression. (**a**) Hierarchical matrix reflecting pairwise comparisons in factor abnormality levels. Element *i,j* represents the total percentage of regions and time points at which the biological factor *j* is more abnormal than is the factor *i*. (**b**) Multifactorial temporal ordering in disease progression, based on the factor-specific abnormality trajectories (temporal abnormalities averaged across all brain regions), memory deficit and three CSF biomarkers (Aβ_1−42_, tau and ptau). All of the results were calculated for the HC to LOAD clinical transition. Dotted lines indicate 95 % confidence intervals, reflecting the uncertainties associated to the estimated mean trajectories, and obtained with 500 bootstrapping resamples. Inset figure provides detail of the trajectories obtained for early states of the disease (HC to EMCI transition). Note how in the initial states the vascular component is separating from the other components, while Aβ, metabolism and functional dysregulation remain close, with a notable overlap among their confidence intervals, until more advanced pathological states. See Supplementary Fig. 2 for equivalent results obtained evaluating the model assuming a sigmoid (instead of linear) relationship between age and disease state, respectively.

First, we did a clinical pairwise comparison between all imaging biomarkers, based on their reconstructed spatiotemporal abnormalities. For each pair of factors (imaging modalities), and for each brain region and time point, a value of 1 was assigned to the factor with the higher abnormality value. This comparison was repeated across all brain regions and time points, and the results were summarized in a 5 × 5 hierarchical matrix (Fig. 3a). Each element *i,j* (*i,j*=1..5) of this hierarchical matrix reflects the percentage of regions and time points at which the imaging modality *j* exceeded in abnormality magnitude the modality *i* during the HC to LOAD clinical transition. The columns of the matrix were reordered keeping from left to right the factors predominating in effect levels. We observed a remarkable predominance of the vascular dysregulation component over the other pathologic biomarkers (Fig. 3a). In total, the vascular factor was ∼80% more abnormal across all brain regions and time points than were the other factors considered. It was followed in spatiotemporal abnormality levels by Aβ deposition, metabolic dysfunction, functional impairment and grey matter atrophy.

**Figure 4:**
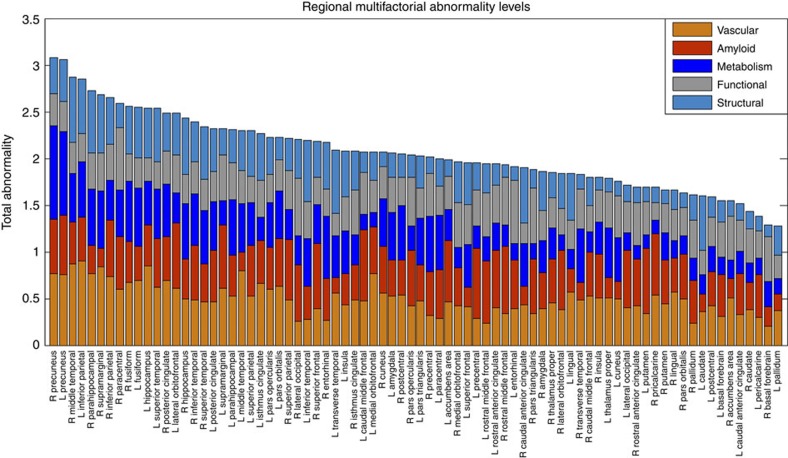
Regional total abnormality levels associated with LOAD progression. Brain regions were sorted from maximum to minimum total effect values, to illustrate their multifactorial damage. Note the across-brain consistent change in the vascular component, which is considerably less prominent for other factors (for example, functional and structural alterations).

Next, to create and compare factor-specific abnormality curves during LOAD development, for each biological factor we calculated the average abnormality curve across all brain regions and, after normalizing by the maximum abnormality value, depicted together the final average curves (Fig. 3b). In the averaging calculation, each region's multifactorial abnormality curves were weighted according to the region's relevance during the pathological progression. For this, we assumed the sum of each region's abnormality levels across all biological factors to be a local multifactorial measure of vulnerability to the disease (Fig. 4). With the purpose of also analysing symptoms severity and peripheral protein alterations as a function of disease progression, we included in our multifactorial analysis the abnormality trajectory obtained for MMSE and for three commonly referenced CSF proteins (Aβ_1–42_, tau and ptau^10^). Again, we observed (Fig. 3b) a higher abnormality magnitude for the vascular component, which exceeded the alterations in the other factors. Consistent with the previous hierarchical results (Fig. 3a), the vascular dysregulation was followed in abnormality magnitude by Aβ deposition, metabolic dysfunction, functional impairment and grey matter atrophy. We noticed similar abnormality levels for Aβ deposition, glucose metabolism and neuronal function at the early stages of the disease. However, these three factors diverged in abnormality levels with disease progression, explaining the global differences observed in the hierarchical matrix (Fig. 3a), and coinciding also with a ‘slower' but consistent increase in structural atrophy. Around the last phase of the LMCI period, the structural atrophy becomes more abnormal (in terms of biomarker distance to the healthy state) than the functional impairment. In addition, we observed symptoms of memory impairment from very early disease stages. Contrary to what previous observational models proposed^9^,^10^, alterations in memory preceded the abnormalities observed for different molecular biomarkers (for example, CSF Aβ_1–42_, tau and ptau proteins). This suggests that cognitive decline associated with LOAD is not a final output of large brain changes, but a continuous consequence of subtle pathological alterations in primary disease factors (for example, vascular dysregulation and Aβ effects).

See Discussion section for further biological interpretation of these results.

### Peripheral vascular and inflammatory alterations

**Figure 5:**
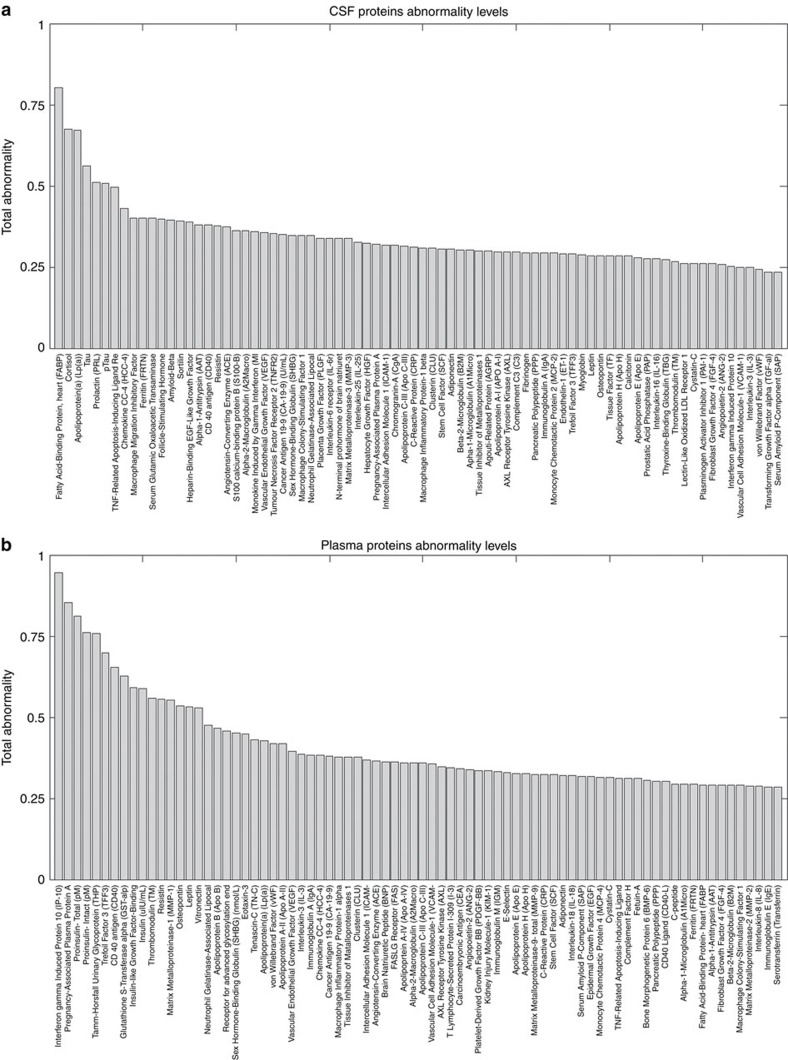
Total CSF and plasma biomarkers abnormality levels associated with LOAD progression. Total CSF (**a**) and plasma (**b**) biomarkers abnormality levels associated with LOAD progression. For detailed lists of biospecimens and the obtained abnormality values for intermediate disease states, see Supplementary Tables 2 and 3.

Proteins execute central functions in living organisms and their peripheral concentrations/interactions are strongly associated with individual health conditions. This makes the analysis of peripheral protein dynamics a crucial step towards understanding the biological mechanisms underlying aging and associated neurodegenerative diseases. Particularly, peripheral plasma and CSF protein measurements have been suggested as promising biomarkers of pre-symptomatic pathological processes underlying LOAD^18^,^19^. Here similar to the imaging biomarkers, we aimed to explore possible abnormalities in plasma and CSF proteins associated with LOAD progression. For this, 146 plasma and 87 CSF protein biomarkers were analysed and sorted according to their obtained LOAD–abnormality indices (Fig. 5; Supplementary Tables 2 and 3).

**Figure 6:**
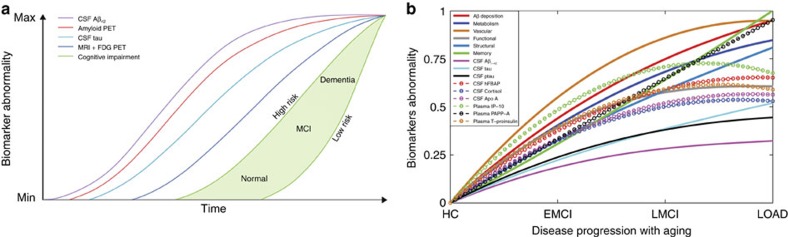
Hypothetical and data-driven models of LOAD progression. Hypothetical (**a**) and data-driven (**b**) models of LOAD progression. (**a**) Adapted from Jack *et al*.^10^, (with permission from Elsevier). (**b**) On the basis of our statistical analysis (Results section, Figs 2, 3, 4, 5, Supplementary Tables 2 and 3). Confidence intervals were omitted for visual clarity. Crucial inter-model differences are: (1) the absence of a vascular component in **a** and the subsequent assumption that Aβ measurements are the earliest biomarkers, whereas in **b** the vascular dysfunction is the earliest/stronger altered event, followed by Aβ deposition; (2) CSF Aβ_42_ and tau are proposed in **a** as the two major proteinopathies underlying LOAD, with higher sensitivity to disease progression than the metabolic/structural and memory biomarkers, however our results suggest that these proteins were not the strongest altered CSF proteins during disease progression (for example, plasma IP-10, PAPP-A and total proinsulin, and CSF hFABP, cortisol and Apo A, showed higher sensitivity) while imaging and memory biomarkers appeared consistently as earlier biomarkers (see Results section, and Supplementary Tables 2 and 3); (3) in **a**, abnormalities in cognitive decline are only detectable at advanced abnormality levels for the considered biological biomarkers. In contrast, in **b**, alterations in cognition are observable in parallel with changes in the primary disease factors (for example, vascular/metabolic dysfunction and Aβ deposition) and exceed in magnitude alterations observed for CSF Aβ_1−42_, tau and ptau.

Heart-type fatty acid-binding protein (hFABP) was identified as the most abnormal CSF biospecimen (Fig. 5a,b, Supplementary Table 3). CSF hFABP levels are known to be significantly altered in LOAD patients^20^, having a high predictive power of the progression from MCI to LOAD states^20^,^21^. The CSF hFABP level is significantly associated with longitudinal atrophy of the entorhinal cortex and other LOAD-vulnerable neuroanatomical regions^22^, and is also considered a sensitive biomarker of specific cardiovascular disorders^23^. Cortisol and Apolipoprotein A (Apo A) were identified as the other most abnormal CSF biospecimens (Fig. 5a). Cortisol is a relevant risk factor for stress, glucose and cardiovascular dysregulation^24^, which has been strongly linked to early phases of LOAD progression and to the hyperactivity of the hypothalamic-pituitary-adrenal axis^25^. Apo A is a high-density lipoprotein with a central role in lipid metabolism. Peripheral Apo A concentration is strongly associated with the integrity of the vascular system and the risk of developing cardiovascular disorders^26^. In addition, we observed high abnormality levels for other CSF measurements previously associated with LOAD progression, for example, tau, ptau, ferritin and Aβ_1–42_ (scored in the total LOAD–abnormality positions 4, 6, 10 and 13, respectively, out of all of the 87 considered CSF biomarkers). However, and contrary to what has been suggested by previous LOAD models^9^,^10^ (Fig. 6a), alterations observed in Aβ_1-42_ proteins were considerably lower than those observed for other CSF proteins (for example, hFBAP, cortisol and Apo A, with approximately a twofold higher average abnormality level for these latest descriptors). This effect was consistent from early to advanced disease states, which might suggest that hFBAP, cortisol and Apo A protein levels in the CSF could be earlier LOAD biomarkers than Aβ_1–42_ concentration.

Among all of the studied plasma biospecimens, interferon-γ-induced protein 10 (IP-10) presented the highest abnormality levels (Figs 5a and 6b, Supplementary Table 2). Alterations in plasma IP-10 reflect peripheral inflammation processes, which are a characteristic feature in aging and associated neurodegenerative disorders^27^. Among other functions, IP-10 is a strong modulator of angiogenesis^28^, which has a key role in poor vascularization and abnormal vasculature disorders^29^. IP-10 was followed in total abnormality levels by pregnancy-associated plasma protein A (PAPP-A), a predictor of adverse vascular events, including high risk of heart infarction^30^. Total and intact proinsulin followed IP-10 and PAPP-A in plasma abnormality levels. Proinsulin is the main precursor of insulin (scored at position 10 out of all the 146 plasma biomarkers). The consistent alterations of insulin and associated proteins in LOAD, and the presence of common cellular responses and pathogenesis, have motivated the classification of this disorder as a form of type III diabetes^31^. Peripheral insulin is suggested to enter the brain via a saturation mechanism involving the blood–brain barrier (BBB)^32^. Alterations in BBB permeability, which recently have been observed at early stages of LOAD^33^, might be associated with alterations in brain insulin resistance^34^. Moreover, peripheral and brain insulin alterations may alter the BBB transport of amino acids and drugs^32^, as well as induce changes in brain glucose, Aβ and ptau regulations^35^. Glutathione S-transferase alpha and plasma matrix metalloproteinase 1 (MMP1) proteins were also identified with high abnormality levels. Glutathione S-transferase alpha alterations are strongly associated with oxidative stress^36^, which is caused by the age-dependent imbalance between the generation and detoxification of reactive oxygen and nitrogen species^37^. Among other relevant pathogenic functions, oxidative stress constitutes a regular pathway for different brain mechanisms leading to BBB dysfunction^38^. Brain MMP1 concentrations have been found to be significantly elevated in LOAD subjects^39^. Matrix metalloproteinase alterations are thought to be linked to neuroinflammatory processes^40^,^41^ and BBB dysfunction^39^,^41^. In summary, changes of these plasma biomarkers suggest an early alteration of the peripheral vascular system during LOAD progression, as well as allude to other relevant pathologic mechanisms (for example, inflammatory hyperactivation).

## Discussion

Neurodegenerative disorders are the consequence of aging-associated multifactorial biological dysfunction. Sophisticated modelling of spatiotemporal abnormalities associated with LOAD progression is a crucial step towards the understanding of the pathological mechanisms underlying this disease, and possibly contributing to the development of effective, disease-modifying therapies. Here we reconstructed LOAD–abnormality trajectories for multiple *in vivo* brain and peripheral biological descriptors. Under the assumption that these biomarkers represent specific physiological processes, we obtained, for the first time to our knowledge, an integrative data-driven model of LOAD progression. In general, our results suggest the role of vascular dysregulation on the early cascade of events associated with the disease's progression (Figs 3, 4, 5, 6, Supplementary Tables 2 and 3).

Due to the increasing high prevalence of LOAD and other neurodegenerative diseases, it is imperative to define realistic biomarker-based models of disease progression^42^. Compared with traditional observational disease models^9^,^10^ (see Fig. 6 for a comparison with our results), data-driven models are less susceptible to subjective expert criteria, as the conclusions are dictated by real data values^11^,^12^,^13^,^14^,^17^. Also, and importantly, data-driven analyses allow for adding and interrelating large amounts of diverse/complementary data (see refs 14, 17). For example, in this study we summarized in a comprehensive framework the information contained in five different brain imaging modalities and tens of peripheral biospecimens. Previous generative models of LOAD progression were notably valuable for understanding the underlying pathological mechanisms^11^,^12^,^13^,^43^,^44^. These models offered spatiotemporal descriptions of the amyloid, metabolic and/or structural changes associated with LOAD. Here we extended such models to capture disease-related changes in other relevant imaging (that is, vascular, functional) and protein (that is, plasma, CSF) descriptors. This broad extension allowed us to obtain an integrative multifactorial description of the disease's progression, characterizing significant pathological alterations from the molecular to the macroscopic scales. A quantitative comparison between the mentioned data-driven models^11^,^12^,^13^,^14^,^17^ is still missing. Due to its importance for recognizing/summarizing methodological differences and to reach a deeper consensus about LOAD progression, it will be among the objectives of our future research.

The comparison between unhealthy and healthy aging and the generation of temporal abnormality trajectories are crucial steps in our progression analysis. First, time plays a central role in the causal cascade of events that contribute to adverse clinical symptoms. Also, progressive neurodegenerative diseases can temporally coexist with non-pathological aging effects, making age a major confounding factor in biomarker examinations^45^. Findings obtained at a given cross-sectional point do not necessarily represent the dynamics of abnormal events associated with a large pathological progression, neither are they associated with time-dependent multifactorial pathological interrelations. Thus, in addition to its multifactorial attribute, the temporal aspect of the proposed generative analysis implies key advantages over traditional neurodegenerative cross-sectional studies. Methodologically, this study provides important contributions towards the integration of multi-modal data sets (for example, bridging neuroimaging and molecular fields) and its application in the study of aging and neurodegenerative disorders.

The causal role of vascular dysregulation on LOAD has been suggested from the beginning of the 20th century (for a recent review, see ref. 3). Although largely undervalued under the rise of other recent hypotheses (for example, the amyloid cascade hypothesis), a growing body of evidence supports the idea that vascular dysregulation is a major risk factor for LOAD development^3^,^4^,^5^. For instance, a significant age-dependent BBB permeability breakdown, that correlates with cognitive dysfunction, has been observed in the human hippocampus^33^. This aging effect is thought to have a key impact on BBB-mediated misfolded protein clearance and deposition, and consequently on associated misfolded protein toxic effects. In line with this, our results (Figs 3, 4, 5, 6, Supplementary Tables 2 and 3) suggest that vascular dysregulation is an early pathological event during disease development, followed in biomarker changing levels by Aβ deposition, metabolic dysfunction, functional impairment and structural atrophy. Although our MFDDA does not reveal causal pathologic interactions, concordant evidence suggests that in LOAD Aβ deposition is mainly caused by a deficiency in the Aβ clearance system rather than by an Aβ overproduction^43^,^46^, whereas Aβ clearance is associated with the vascular system's integrity^47^,^48^,^49^. Aβ efflux across the BBB sequestrates around 60% of the brain's Aβ proteins^48^. At the same time, Aβ has vascular destructive activity, making the cerebrovasculature a primary target for Aβ toxicity^50^. Also, Aβ is thought to have a negative impact on mitochondrial function, which consequently may increase reactive oxygen species (ROS) production and reduce mitochondrial Aβ clearance, in a continuous feed-forward mechanism^51^. While a decrease in energy availability may affect cellular activity and lead to brain functional impairment, the neurodegenerative progression may decrease glucose metabolism because of the reduced synaptic energy needs^52^. All together, vascular dysregulation, Aβ toxicity, failure in cellular energy demands (that is, hypometabolism) and functional toxicity may cause neuronal/glial cell death (that is, structural atrophy) and cognitive decline in a continuous degenerative cycle.

Importantly, multifactorial pathological interactions are not restricted to local/regional levels. From the microscopic to the macroscopic scales, multimodal brain connections can also be a conduit for disease spreading mechanisms (for review see ref. 52). In addition to contributing to the intercellular transference of factor-specific abnormalities (e.g. propagation of neuronal/synaptic toxicity across anatomical/functional connections), strong relationships persist among the different forms of brain connectivity. For example, the vascular system supplies oxygen, glucose and other nutrients, and clears away deoxygenated blood and metabolic products, having a direct impact on the brain's functional/metabolic activity^53^. However, in a feedback relationship, drastic changes in functional/metabolic activities can notably modulate brain vascular networks^54^. This connectivity-mediated effect can explain spatial mismatches between factor abnormalities observed during disease progression. For example, while regional metabolic alterations in LOAD have been found to follow Aβ deposition in many brain regions^55^, the remaining spatial mismatch between hypometabolism and Aβ binding could be explained by functional connections to Aβ deposition areas^56^,^57^.

In addition to highlighting possible peripheral vascular and inflammatory alterations during LOAD progression, our plasma and CSF analysis suggests possible mechanisms contributing to such dysregulations. Peripheral insulin resistance, inflammatory and lipid/fatty acid metabolism alterations, supported by the observed high proinsulin, IP-10, hFABP and Apo A abnormalities, were some of the main pathological mechanisms suggested by the proteomics findings. Some of these have been previously associated with vascular/metabolic integrity and neurodegenerative progression^3^,^31^,^34^,^35^. Potentially, they might be reflecting the cascade of multifactorial pathological events conducive to LOAD. However, as discussed below, we should be cautious about the interpretation of these findings, since our analysis does not reveal direct causal relationships among the considered biomarkers, and consequently, neither among their corresponding biological factors.

In general, these results could contribute to the development of efficient, cost-effective, therapeutic interventions. The proposed MFDDA could be employed to enrich clinical trial populations by allowing for selective enrolment of subjects at a particular pathological disease transition. In this sense, the availability of characteristic abnormality trajectories for different biomarkers can help to evaluate if a given subject with a specific demographic profile is closest (in terms of biomarkers abnormalities) to one (for example, HC to EMCI) or another possible (for example, LMCI to LOAD) clinical transition. The multivariate distances to the reconstructed characteristic trajectories could represent useful quantitative indices of individual disease transitions. Moreover, and importantly, this model could be employed as an accurate quantitative descriptor of drug response, characterizing and predicting future deviations from characteristic disease trajectories generated with and without considering drug effects. The study of patients under a specific medication could allow to obtain biomarker trajectories as a function of age, disease state and medication levels. When a new patient would be analysed, consistent deviations from a specific trajectory and/or closeness to others could be reflecting how well that particular patient is responding to therapy.

In line with previous models of LOAD progression^9^,^10^,^11^,^12^,^13^, a strong assumption in our study is that the analysed biomarkers precisely reflect specific pathophysiological processes. Qualitative and data-driven models depend on how realistically the available observations represent the underlying biological processes. Although grey matter density and atrophy measurements, obtained with structural MRI techniques, are commonly used to characterize structural brain properties, results and interpretations depend on how accurately the used MRI techniques reflect the real tissue properties and also under which spatial scales these measurements are precise. Similarly, current ASL and PET techniques still offer a limited characterization of the vascular and metabolic/Aβ brain properties. Consequently, it is important to exert caution about the observed biomarkers ordering with disease progression. Although the obtained abnormality trajectories may be reflecting a tentative ordering in which pathophysiological events occur, our results should be interpreted more in terms of biomarker sensitivity to disease progression than in terms of causal pathologic interactions conducive of LOAD. In addition, here structural alterations were only evaluated in the grey matter, ignoring possible alterations within the white matter and in its associated structural connectivity patterns. This will be the main focus of a separate study, for which we are combining structural T1 atrophy and diffusion-weighted connectivity measurements^58^,^59^. Another potential limitation of our study is that all evaluations were performed within a linear regression framework. This could mean that the obtained results are mainly reflecting the linear tendencies in the analysed biomarkers. The alternative use of non-linear modelling techniques (for example, radial basis and kernel functions) may provide a solution to overcome this particular limitation. Similarly, the assumption of an explicit analytic expression associated with each biomarker (for example, equation 3, Methods section) is a limitation in line with some previous data-driven models^13^,^14^,^17^. An event-based perspective^11^,^12^ presents the advantage of not assuming any a priori biomarkers shape. However, the latest models demand a high computational cost to test exhaustively all the possible combinations in events ordering, which can make it difficult to apply such perspectives to a high number of multifactorial biomarkers. Finally, and similarly to the previous models^9^,^10^,^11^,^12^,^13^,^14^, here we are not addressing the issue that initial small changes/alterations in specific biological factors could potentially cause large alterations in other interconnected factors. This traditional limitation suggests the need to study disease progression not only in terms of alteration levels of specific biomarkers, but also through the analysis of the multifactorial causal pathological interactions that take place at the different spatiotemporal scales. Causal analyses could potentially lead us to a more integrative understanding of neurodegenerative progression, and will form the central purpose of our future research.

## Methods

### Ethics statement

The study was conducted according to Good Clinical Practice guidelines, the Declaration of Helsinki Principles, US 21CFR Part 50—Protection of Human Subjects, and Part 56—Institutional Review Boards, and pursuant to state and federal HIPAA regulations (adni.loni.usc.edu). Study subjects and/or authorized representatives gave written informed consent at the time of enrolment for sample collection and completed questionnaires approved by each participating sites Institutional Review Board. The authors obtained approval from the ADNI Data Sharing and Publications Committee for data use and publication, see documents http://adni.loni.usc.edu/wp-content/uploads/how_to_apply/ADNI_Data_Use_Agreement.pdf and http://adni.loni.usc.edu/wp-content/uploads/how_to_apply/ADNI_Manuscript_Citations.pdf, respectively.

### MFDDA of LOAD progression

Here we considered that LOAD progression can be characterized by a set of *N* biological and behavioural descriptors/biomarkers, where each descriptor *i* (*i=1..N*) is uniquely described by its temporal abnormality level (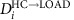) and a total abnormality index (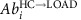). As aging is a major risk factor for LOAD, the dynamic behaviour of the abnormality level should depend on the statistical distance between an unhealthy (LOAD associated) and a healthy aging:


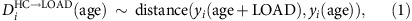


where *y*_*i*_ is the spatiotemporal function describing the dynamics of the biomarker *i* during aging, including or not the influence of pathological factors. In addition to age and clinical state, we considered gender (gen), educational level (edu) and apoe-e4 genotype (*apoeɛ4*) as other aging and disease relevant factors. Note that the inclusion of these variables here should not be considered exclusive of other possible risk factors in posterior LOAD studies (for example, familial history, life style conditions). Then, each biomarker observation can be written as:


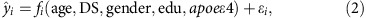


where *f*_*i*_ is a function interrelating the risk factors and biomarker *i*, DS is the individual disease state, reflecting LOAD progression and symptoms severity, and *ɛ*_*i*_ is a noise term capturing the individual variability associated to *i*. For the sake of simplicity on model definition and evaluation, here we considered each *f*_*i*_ as an additive linear function, assuming the previous variables and all their possible pairwise interactions (that is, 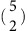
=10 interactions) as predictors, and considering also possible random effects across patient visits:


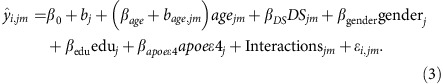



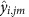
 is the estimator of the biomarker *i* for the subject *j* at time visit *m*. All β coefficients in (3) correspond to fixed effects across the entire population, whereas *b*_*j*_ and *b*_*age,jm*_ coefficients correspond to random effects modelling longitudinal changes within each subject *j*. For each biomarker, we used the *Bayesian Information Criterion* (BIC) to select among the three possible models associated to expression (3): (i) a purely fixed effects model (i.e. *b*_*j*_=*b*_age*,jm*_=0), (ii) a mixed effects model with different intercepts and fixed slopes (i.e. *b*_*j*_≠0,*b*_age*,jm*_=0) or (iii) a mixed effects model with different time intercepts and different slopes (i.e. *b*_*j*_≠0,*b*_age*,jm*_≠0). Parameters estimation for alternative (i) was performed using a robust linear regression algorithm^60^, whereas estimations for (ii) and (iii) were based on the Fisher Scoring optimization^61^. Before estimations, all biomarkers were standardized to have mean 0 and s.d. 1 across subjects.

As individual DS measure, here we used the individual clinical diagnoses assigned by the ADNI experts, which were based on multiple clinical evaluations. The assumed DS values ranged from 1 to 4, with DS=1 (HC), DS=2 (EMCI), DS=3 (LMCI) and DS=4 (LOAD), respectively. Due to the impact of uncontrolled factors during data acquisition and cognitive/clinical evaluations (for example, pre-symptomatic effects and noisy biomarkers measurements), the raw data can present a given level of heterogeneity inside each clinical group^11^. Thus, to control for possible heterogeneity effects, before model fitting and evaluations we performed a robust data homogeneity/quality control, consisting of three main steps (see Supplementary Note 1):

- Identification and elimination of all subjects that presented clinical conversions, across the whole dataset acquisition.
- Calculation of individual likelihood scores reflecting how accurately each subject was diagnosed by the clinical experts, and subsequent elimination of the subjects with low likelihood scores (below the 10th percentile). For further details, see Supplementary Note 1.
- For each biomarker and clinical group, outlier identification was performed based on the Mahalanobis distance, with a significative squared distance (*P*<0.05) meaning an outlier (for implementation details, see ref. 62). Outlier detection for imaging biomarkers considered all brain regions, using the multivariate Mahalanobis distance.

Both steps (i) and (ii) controlled for cognitive heterogeneity, whereas (iii) controlled for variability and noise on biological measurements, improving together cognitive and biological data homogeneity at the clinical group levels. Importantly, although the choice of a categorical DS variable may overlook possible individual variability beyond the clinical classification, a strong correlation has been observed between the clinical diagnoses within ADNI data and a continuous LOAD disease progression score obtained after integrating multiple cognitive, neuroimaging and biospecimen biomarkers^17^.

Once the parameters for each *f*_*i*_ expression were estimated based on the homogeneity/quality controlled data, we marginalized the *f*_*i*_ expression by gender, educational level and apoe-e4, from their minimum to their maximum values, respectively (for mathematic details, see Supplementary Note 1). This is equivalent to keeping only the temporal (age) and disease components (DS) in a final 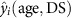 function, after weighting by the effects of the marginalized risk factors. Note that such weighting method differs notably from the traditional covariate ‘controlling' procedure (that is, removing the covariate effects on each descriptor^13^), which may cause the loss of relevant risk factor effects on the disease's analysis and the deletion of useful information contained in the data.

Next, using the marginalized estimator 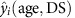, each descriptor was analytically reconstructed under different aging and disease conditions. For this, different possible aging and disease trajectories were generated, covering all the possible clinical state transitions during aging, with the DS going from: HC to HC, HC to EMCI, HC to LMCI, and HC to LOAD states, respectively. Similar to the approach described in ref. 13, the generative procedure consisted in evaluating the marginalized 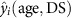 for different values of age and DS (Supplementary Fig. 1). For example, for a HC to LOAD transition, ages took on 1560 increasing values (that is, once every week) while the DS incremented linearly with age (or following a sigmoid, reaching similar conclusions; Supplementary Figs 1 and 2) from the typical values for the HC subjects (DS=1) to the typical values for LOAD subjects (DS=4). Then, at each age/disease time point, the dynamic abnormality level for the characteristic HC to LOAD trajectory was calculated as its absolute difference with the same time point on the HC to HC trajectory^13^:


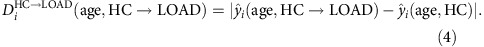


This expression (4) reflects quantitatively how, due to the disease progression, a given biomarker *i* differentiates from its age-matched values at the healthy stage. Importantly, as all the descriptors were previously standardized (that is, to have mean 0 and s.d. 1), the values obtained from (4) allow the comparison (in terms of absolute distance to a healthy aging) of the alterations occurring to the different biological descriptors/biomarkers. Subsequently, and based on this distance/abnormality metric (4), for each descriptor *i* a total abnormality index (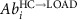 ɛ [0, 1]) was evaluated, summarizing its normalized dissimilarity with healthy aging across the entire disease/aging progression interval, in the HC to LOAD trajectory:


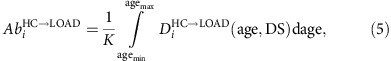


where *K* is a normalization constant, guaranteeing a maximum total abnormality index of 1 across all considered biomarkers. Potentially, some biomarkers can be abnormal at early disease states and tend to have normal values with aging and/or advanced disease progression processes (for example, a protein with an increase in concentration for early disease states, and a posterior concentration decrease due to a down regulation in its associated gene). In such cases the total abnormality index (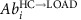) could give a biased measure of how early these biomarkers become affected. To overcome this we also calculated intermediate abnormality indexes (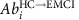 and 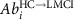, respectively), evaluating expression (5) until the corresponding EMCI and LMCI time points were reached (see Supplementary Tables 2 and 3).

Finally, for each biomarker, we used a bootstrapping procedure (creating 500 different data sets with replacement) to calculate the mean and the 95 % confidence intervals of the obtained characteristic trajectories and their associated abnormality levels (see Fig. 3). All the results reported (Figs 2, 3, 4, 5, 6 and Supplementary Tables 2–3) and associated biological interpretations in this study were based on the mean bootstrapped outcomes.

For algorithmic details, see Supplementary Note 1.

### Data description and processing

*Study participants*. This study used 1,171 individual data from ADNI (adni.loni.usc.edu). The ADNI was launched in 2003 as a public-private partnership, led by Principal Investigator Michael W. Weiner, MD. The primary goal of ADNI has been to test whether serial MRI, PET, other biological markers, and clinical and neuropsychological assessment can be combined to measure the progression of MCI and early Alzheimer's disease.

See Supplementary Table 1 for demographic characteristics of the included ADNI subjects.

*Structural MRI acquisition/processing*. Brain structural T1-weighted three-dimensional images were acquired for all subjects. For a detailed description of acquisition details, see http://adni.loni.usc.edu/methods/documents/mri-protocols/. All images underwent non-uniformity correction using the N3 algorithm^63^. Next, they were segmented in grey matter, white matter and CSF probabilistic maps, using SPM12 (www.fil.ion.ucl.ac.uk/spm). Grey matter segmentations were standardized to MNI space using DARTEL tool from SPM12. Each map was modulated to preserve the total amount of signal/tissue. Mean grey matter density and determinant of the Jacobian (DJ) values were calculated for 78 regions covering all the brain's grey matter^15^. For each region, obtained density and DJ values were statistically controlled for differences in acquisition protocols. Both measurements provided equivalent modelling results. All the results/figures presented in this study correspond to the DJ, which constitutes a robust local measure of structural atrophy.

*Fluorodeoxyglucose PET acquisition/processing*. A 185 MBq (5+0.5 mCi) of [18F]-FDG was administered to each participant and brain PET imaging data were acquired ∼20 min post injection. All images were corrected using measured attenuation. Also, images were preprocessed according to four main steps^64^: (1) dynamic co-registration (separate frames were co-registered to one another lessening the effects of patient motion), (2) across time averaging, (3) re-sampling and reorientation from native space to a standard voxel image grid space (‘AC-PC' space) and (4) spatial filtering to produce images of a uniform isotropic resolution of 8 mm FWHM. Next, using the registration parameters obtained for the structural T1 image with nearest acquisition date, all FDG-PET images were spatially normalized to the MNI space. Regional standardized uptake value ratio (SUVR) values for the considered 78 regions^15^ were calculated taking the cerebellum as reference region.

*Resting functional MRI acquisition/processing*. Resting-state functional images were obtained using an echo-planar imaging sequence, on a 3.0-Tesla Philips MRI scanner. Acquisition parameters were: 140 time points, repetition time (TR)=3,000 ms, echo time (TE)=30 ms, flip angle=80°, number of slices=48, slice thickness=3.3 mm, spatial resolution=3 × 3 × 3 mm^3^ and in plane matrix = 64 × 64. Preprocessing steps included: (1) motion correction, (2) slice timing correction, (3) spatial normalization to MNI space using the registration parameters obtained for the structural T1 image with nearest acquisition date, and (4) signal filtering to keep only low-frequency fluctuations (0.01–0.08 Hz)^65^. To have regional quantitative indicators of the brain's functional integrity, fractional amplitude of low-frequency fluctuation^66^, regional homogeneity^67^ and functional connectivity degree^68^ measures were calculated for each considered brain region. Among these three measurements, low-frequency fluctuation showed the highest sensitivity to disease progression. Consequently, all the results presented in this study correspond to this measure.

*ASL acquisition/processing*. Resting ASL data were acquired using the Siemens product PICORE sequence. Acquisition parameters were: TR/TE=3,400/12 ms, TI1/TI2=700/1,900 ms, FOV=256 mm, 24 sequential 4 mm thick slices with a 25% gap between the adjacent slices, partial Fourier factor=6/8, bandwidth=2,368 Hz/pix, and imaging matrix=64 × 64. For preprocessing details see ‘UCSF ASL Perfusion Processing Methods' in www.adni.loni.usc.edu. In summary, main preprocessing steps included: (1) motion correction, (2) perfusion-weighted images (PWI) computation, (3) intensity scaling, (4) cerebral blood flow (CBF) images calculation, (5) spatial normalization to MNI space using the registration parameters obtained for the structural T1 image with nearest acquisition date and (6) mean CBF calculation for each considered brain region.

*Aβ PET acquisition/processing*. A 370 MBq (10mCi±10%) bolus injection of AV-45 was administered to each participant, and 20 min continuous brain PET imaging scans were acquired ∼50 min post injection. The images were reconstructed immediately after the 20-min scan, and when motion artifact was detected, another 20-min continuous scan was acquired. For each individual PET acquisition, images were initially preprocessed according to four main steps^64^: (1) dynamic co-registration (separate frames were co-registered to one another lessening the effects of patient motion), (2) across time averaging, (3) re-sampling and reorientation from native space to a standard voxel image grid space (“AC-PC” space), and (4) spatial filtering to produce images of a uniform isotropic resolution of 8 mm FWHM. Next, using the registration parameters obtained for the structural T1 image with nearest acquisition date, all amyloid images were spatially normalized to the MNI space. Considering the Cerebellum as an Aβ non-specific binding reference, regional SUVR values for the considered 78 grey matter regions were calculated.

*Plasma measures*. Levels of 146 analytes (Supplementary Table 2) were measured from subject plasma, using the rules-based medicine (rulesbasedmedicine.com, Austin, TX) Human Discovery Multi-Analyte Profile (MAP) 1.0 panel and a Luminex 100 platform. For further acquisition and preprocessing details see ref. 69 and http://adni.loni.usc.edu/wp-content/uploads/2010/11/BC_Plasma_Proteomics_Data_Primer.pdf.

*CSF measures*. Levels of 87 analytes (Supplementary Table 3) were measured from subject CSF, using a multiplex-based immunoassay panel based on Luminex immunoassay technology developed by rules-based medicine (MyriadRBM, Austin, TX, USA). For further acquisition and preprocessing details see http://adni.loni.usc.edu/wp-content/uploads/2012/01/2011Dec28-Biomarkers-Consortium-Data-Primer-FINAL1.pdf.

### Data availability

All data used in this study is available at the Alzheimer's Disease Neuroimaging Initiative (ADNI) database (adni.loni.usc.edu).

## Additional information

**How to cite this article:** Iturria-Medina, Y. *et al*. Early Role of Vascular Dysregulation on Late-Onset Alzheimer's Disease based on multifactorial data-driven analysis. *Nat. Commun.* 7:11934 doi: 10.1038/ncomms11934 (2016).

## Supplementary Material

Supplementary Information Supplementary Figure 1-2, Supplementary Tables 1-3, Supplementary Note 1 and Supplementary References.
