# Supplementary material for: Early role of vascular dysregulation on late-onset Alzheimer's disease based on multifactorial data-driven analysis
Source: Nat Commun. 2016 Jun 21;7:11934. doi: 10.1038/ncomms11934 (PMC4919512; doi:10.1038/ncomms11934)
Supplement: Supplementary Information — Supplementary Figure 1-2, Supplementary Tables 1-3, Supplementary Note 1 and Supplementary References. [file ncomms11934-s1.pdf]

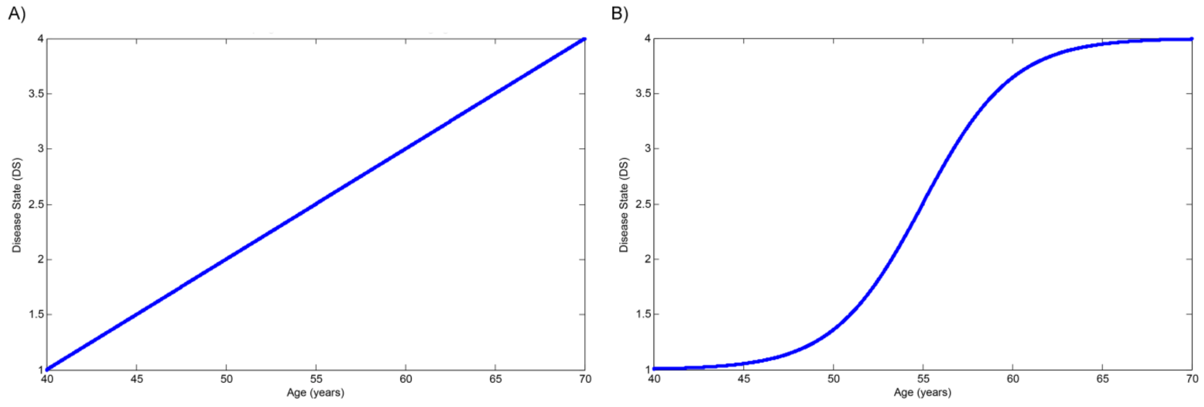

Supplementary Fig. 1. Assumed disease state (*DS*) vs aging relationships. In a) the *DS* increases linearly with age, while in b) it follows a sigmoid relationship with age (as suggested by [1]). In both cases, the assumed *DS* values ranged from 1 to 4, with DS=1 (for HC), DS=2 (for EMCI), DS=3 (for LMCI) and DS=4 (for LOAD).

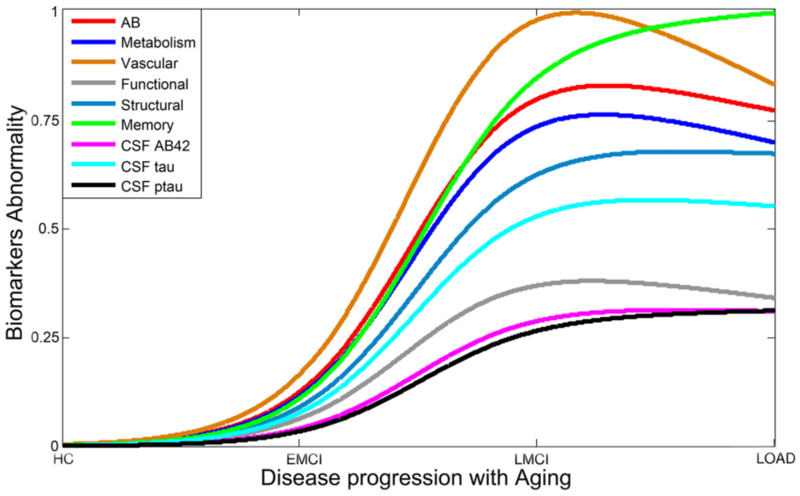

Supplementary Fig. 2. Multi-factorial temporal ordering in disease progression, based on the factors-specific abnormality trajectories (temporal abnormalities averaged across all brain regions) and memory deficit. All results were calculated for the HC to LOAD clinical transition, using a sigmoid (rather than linear) relationship between age and disease state. In general, these results are similar to those obtained following a linear relation. However, there are two relevant differences that, in the context of our model (Equation 3), support the preferential use

of a linear over a sigmoid relationship. First, notice a decrease in vascular, Aβ, metabolic and functional alteration levels around the final period of disease progression. The use of a sigmoid function for *DS* vs age introduces a strong non-linearity effect on the interaction term (*age*DS*) of Equation 3, and eventually, for high values of age and DS, a visual “degeneration” effect on the estimated abnormality trajectories. Notice also that structural abnormalities are now earlier/stronger than functional alterations. In Fig. 3b, we observed that at advanced disease states (around the end of the LMCI phase) the changes in structural biomarkers exceed the functional alterations. The alternative use of a sigmoid function, implying considerably faster changes in disease state with aging, seems to reinforce the previous effect starting from the early ages of the pathologic process.

Supplementary Table 1. Demographic characteristics of the included ADNI subjects.

**Characteristics**	**Aβ PET** (n=907; n_{long}=1079)	**FDG PET** (n=1070; n_{long}=1680)	**ASL** (n=324; n_{long}=1145)	**rfMRI** (n=164; n_{long}=621)	**T1** (n=1092; n_{long}=4943)	**Plasma** (n=1063)	**CSF** (n=326)	**MMSE** (n=1092; n_{long}=5129)
Women	396(44.0%)	479(44.7%)	152(47.0%)	85(51.8%)	486(44.5%)	406(38.1%)	129(39.5%)	486(44.5%)
Age (years)	73.2(7.3)	73.4(7.3)	72.8 (7.3)	72.6(7.1)	73.4(7.3)	75.1(7.3)	74.9(6.9)	73.4(7.3)
APOE e4 (1 copy)	323(35.6%)	383(35.8%)	105(32.4%)	62(37.8%)	387(35.4%)	414(38.9%)	118(36.2%)	385(35.4%)
APOE e4 (2 copies)	85(9.3%)	103(9.6%)	37(11.4%)	19(11.6%)	106(9.7%)	134(12.6%)	39(11.9%)	106(9.7)
Education (years)	16.2(2.7)	16.0(2.7)	16.2(2.71)	16.1(2.6)	16.1(2.7)	15.6(3.0)	15.6(3.0)	16.1(2.7)

MMSE, mini-mental state examination; Data are number (%) or mean (std); N_{long}, samples sizes considering longitudinal measures.

Supplementary Table 2. LOAD-abnormality indices obtained for the PLASMA proteins.

Order	Plasma Proteins	$Ab^{HC\rightarrow EMCI}$	$Ab^{HC\rightarrow LMCI}$	$Ab^{HC\rightarrow LOAD}$
1	Interferon gamma Induced Protein 10 (IP-10)	0.47018605	0.78562206	0.94504416
2	Pregnancy-Associated Plasma Protein A	0.29368219	0.57684934	0.85336554
3	Proinsulin-Total (pM)	0.38899556	0.66011727	0.81213075
4	Proinsulin-Intact (pM)	0.36092117	0.61473215	0.76026428
5	Tamm-Horsfall Urinary Glycoprotein (THP)	0.28809053	0.54038155	0.75729394
6	Trefoil Factor 3 (TFF3)	0.27015981	0.50277877	0.69835812
7	CD 40 antigen (CD40)	0.27538958	0.49276611	0.65195173
8	Glutathione S-Transferase alpha (GST-alp)	0.27274245	0.48176119	0.62779182
9	Insulin-like Growth Factor-Binding	0.31165662	0.50849050	0.59057343
10	Insulin (uIU/mL)	0.27564579	0.47136232	0.58667529
11	Thrombomodulin (TM)	0.20698848	0.39205167	0.55844575
12	Resistin	0.23660634	0.42094636	0.55412018
13	Matrix Metalloproteinase-1 (MMP-1)	0.24334116	0.42786169	0.55281436
14	Osteopontin	0.24598694	0.42356995	0.53276998
15	Leptin	0.28786165	0.46462533	0.53116441
16	Vitronectin	0.20342088	0.37865624	0.52762866
17	Neutrophil Gelatinase-Associated Lipocal	0.17440116	0.33096606	0.47401804
18	Apolipoprotein B (Apo B)	0.20056985	0.35510364	0.46517205
19	Receptor for advanced glycosylation end	0.16168442	0.31027865	0.45685256
20	Sex Hormone-Binding Globulin (SHBG) (nmol/L)	0.16924179	0.31743869	0.45175001
21	Eotaxin-3	0.21554595	0.36458603	0.44667873
22	Tenascin-C (TN-C)	0.19245045	0.33469194	0.43063048
23	Apolipoprotein(a) (Lp(a))	0.17875262	0.31923479	0.42563087
24	von Willebrand Factor (vWF)	0.18482710	0.32415381	0.41772756
25	Apolipoprotein A-II (Apo A-II)	0.20988773	0.34857580	0.41677472
26	Vascular Endothelial Growth Factor (VEGF)	0.15561053	0.28514570	0.39566103
27	Interleukin-3 (IL-3)	0.15240858	0.27906454	0.38563555
28	Immunoglobulin A (IgA)	0.20444877	0.33062690	0.38202426
29	Chemokine CC-4 (HCC-4)	0.19953899	0.32601506	0.38118711
30	Cancer Antigen 19-9 (CA-19-9)	0.17455800	0.30059221	0.37917778
31	Macrophage Inflammatory Protein-1 alpha	0.15211897	0.27372041	0.37714851
32	Tissue Inhibitor of Metalloproteinases 1	0.14382499	0.26701099	0.37692326
33	Clusterin (CLU)	0.13293795	0.25533396	0.37657696
34	Intercellular Adhesion Molecule 1 (ICAM-	0.22620642	0.34441295	0.36899084
35	Angiotensin-Converting Enzyme (ACE)	0.17403951	0.29493567	0.36556023
36	Brain Natriuretic Peptide (BNP)	0.12066551	0.23501173	0.36192587
37	FASLG Receptor (FAS)	0.24012834	0.35505703	0.36042958
38	Apolipoprotein A-IV (Apo A-IV)	0.17940098	0.29772109	0.35801709
39	Alpha-2-Macroglobulin (A2Macro)	0.23409961	0.34599996	0.35756344
40	Apolipoprotein C-III (Apo C-III)	0.14499220	0.26136339	0.35748589
41	Vascular Cell Adhesion Molecule-1 (VCAM-	0.12012064	0.23309627	0.35692126

42	AXL Receptor Tyrosine Kinase (AXL)	0.14605923	0.26002470	0.34611332
43	T Lymphocyte-Secreted Protein I-309 (I-3)	0.14590628	0.25555488	0.34440729
44	Carcinoembryonic Antigen (CEA)	0.13880558	0.25058526	0.34125474
45	Angiopoietin-2 (ANG-2)	0.16205524	0.27423522	0.33885860
46	Platelet-Derived Growth Factor BB (PDGF-BB)	0.17344713	0.28378278	0.33547634
47	Kidney Injury Molecule-1 (KIM-1)	0.12048283	0.22461690	0.33541965
48	Immunoglobulin M (IGM)	0.12164487	0.22266664	0.33230704
49	E-Selectin	0.16046187	0.26971290	0.32948154
50	Apolipoprotein E (Apo E)	0.12163033	0.22567971	0.32661065
51	Apolipoprotein H (Apo H)	0.20312323	0.30708775	0.32462740
52	Matrix Metalloproteinase-9- total (MMP-9)	0.13567871	0.24255751	0.32359782
53	C-Reactive Protein (CRP)	0.15263543	0.25913706	0.32312748
54	Stem Cell Factor (SCF)	0.15864602	0.26508406	0.32298592
55	Adiponectin	0.13763471	0.24319412	0.32047987
56	Interleukin-18 (IL-18)	0.14599238	0.24979562	0.31941906
57	Serum Amyloid P-Component (SAP)	0.17087953	0.27548775	0.31838110
58	Epidermal Growth Factor (EGF)	0.15179038	0.25690886	0.31759033
59	Monocyte Chemotactic Protein 4 (MCP-4)	0.13675691	0.24008277	0.31339011
60	Cystatin-C	0.10400772	0.20052585	0.31330073
61	TNF-Related Apoptosis-Inducing Ligand	0.14987919	0.25278410	0.31190747
62	Complement Factor H	0.14640401	0.24291873	0.31185040
63	Fetuin-A	0.14062598	0.24296631	0.30980930
64	Bone Morphogenetic Protein 6 (BMP-6)	0.094724402	0.17871289	0.30571124
65	Pancreatic Polypeptide (PPP)	0.13827498	0.23818731	0.30212730
66	CD40 Ligand (CD40-L)	0.12788729	0.22652897	0.30126476
67	C-peptide	0.16229308	0.25843173	0.29394892
68	Alpha-1-Microglobulin (A1Micro)	0.12666690	0.22278295	0.29384130
69	Ferritin (FRTN)	0.13864461	0.23513448	0.29204142
70	Fatty Acid-Binding Protein- heart (FABP	0.13074417	0.22532967	0.29162982
71	Alpha-1-Antitrypsin (AAT)	0.11667685	0.19826698	0.29122028
72	Fibroblast Growth Factor 4 (FGF-4)	0.11612145	0.20505531	0.29076073
73	Beta-2-Microglobulin (B2M)	0.10205363	0.18964648	0.29021794
74	Macrophage Colony-Stimulating Factor 1	0.13053468	0.22597143	0.28898633
75	Matrix Metalloproteinase-2 (MMP-2)	0.098336682	0.18791500	0.28635415
76	Interleukin-8 (IL-8)	0.12980977	0.22428279	0.28581798
77	Immunoglobulin E (IgE)	0.12398396	0.21543309	0.28408831
78	Serotransferrin (Transferrin)	0.13785082	0.22990817	0.28340709
79	Apolipoprotein C-I (Apo C-I)	0.13774310	0.23117383	0.28233925
80	Hepatocyte Growth Factor (HGF)	0.16338615	0.24786240	0.27655956
81	Ciliary Neurotrophic Factor (CNTF)	0.16409084	0.25040707	0.27647656
82	Growth-Regulated alpha protein (GRO-alph	0.14005369	0.23121011	0.27581832
83	Tumor Necrosis Factor Receptor-Like 2	0.083985515	0.16622767	0.27066824
84	Complement C3 (C3)	0.13463171	0.21468554	0.27037165
85	Neuronal Cell Adhesion Molecule (Nr-CAM)	0.12507422	0.21427999	0.27034146
86	Tumor Necrosis Factor alpha (TNF-alpha)	0.13365276	0.21024328	0.26971221
87	Pulmonary and Activation-Regulated Chemo	0.14103895	0.21068704	0.26899195

88	Epidermal Growth Factor Receptor (EGFR)	0.11193015	0.19280189	0.26616415
89	Epithelial-Derived Neutrophil-Activating	0.14087620	0.22794294	0.26583767
90	CD5 (CD5L)	0.12905447	0.21438783	0.26341066
91	Prostatic Acid Phosphatase (PAP)	0.10374783	0.18883698	0.26305330
92	Thyroxine-Binding Globulin (TBG)	0.13251136	0.20792723	0.26183227
93	Testosterone- Total	0.11204125	0.19799426	0.25885257
94	Thrombopoietin	0.14008316	0.21652716	0.25666755
95	Monocyte Chemotactic Protein 3 (MCP-3)	0.14495806	0.21810088	0.25118107
96	Interleukin-16 (IL-16)	0.11244825	0.19504246	0.25014463
97	Monokine Induced by Gamma Interferon	0.097977422	0.16780607	0.24798913
98	Angiotensinogen	0.10012230	0.17739354	0.24797095
99	Sortilin	0.11029509	0.18907183	0.24747498
100	Peptide YY (PYY)	0.11831535	0.19534560	0.24745528
101	Transthyretin (TTR)	0.12637106	0.20612295	0.24740542
102	Growth Hormone (GH)	0.10566454	0.18625492	0.24427789
103	Vitamin K-Dependent Protein S (VKDPS)	0.11398441	0.19221938	0.24060434
104	Monocyte Chemotactic Protein 2 (MCP-2)	0.11562925	0.19440697	0.24013631
105	Factor VII	0.11738886	0.19526103	0.23817311
106	Brain-Derived Neurotrophic Factor (BDNF)	0.12446750	0.20135865	0.23722336
107	Macrophage Inflammatory Protein-1 beta	0.10893252	0.18661371	0.23679717
108	Myeloid Progenitor Inhibitory Factor 1	0.10852243	0.18599397	0.23578355
109	Fibrinogen	0.12334146	0.19577144	0.23474562
110	Plasminogen Activator Inhibitor 1	0.10912220	0.18517649	0.23409338
111	Matrix Metalloproteinase-9 (MMP-9)	0.10441891	0.18127251	0.23273608
112	Interleukin-6 receptor (IL-6r)	0.11197791	0.18624486	0.23256527
113	Macrophage Migration Inhibitory Factor	0.10547031	0.18130903	0.23078366
114	Apolipoprotein A-I (Apo A-I)	0.10338872	0.17449750	0.23077528
115	Agouti-Related Protein (AGRP)	0.11322884	0.17475453	0.22841136
116	Eotaxin-1	0.10310928	0.17656523	0.22597176
117	Alpha-Fetoprotein (AFP)	0.11274192	0.18526591	0.22428113
118	Haptoglobin	0.10250797	0.17488678	0.22049826
119	Apolipoprotein D (Apo D)	0.094888933	0.16613102	0.22013372
120	Myeloperoxidase (MPO)	0.091740981	0.16269460	0.21736732
121	B Lymphocyte Chemoattractant (BLC)	0.098744400	0.16900247	0.21611196
122	Macrophage-Derived Chemokine (MDC)	0.10772531	0.17778201	0.21605453
123	Thyroid-Stimulating Hormone (TSH)	0.099158436	0.16814062	0.21218024
124	Chromogranin-A (CgA)	0.10853631	0.17556612	0.21176389
125	Alpha-1-Antichymotrypsin (AACT)	0.093325093	0.16252363	0.21152192
126	Prolactin (PRL)	0.096048661	0.16302630	0.21028547
127	Creatine Kinase-MB (CK-MB)	0.10025492	0.16843008	0.20844321
128	Superoxide Dismutase 1- Soluble (SOD-1)	0.10308488	0.17077239	0.20601930
129	T-Cell-Specific Protein RANTES (RANTES)	0.10044890	0.16738941	0.20494924
130	Myoglobin	0.098138399	0.16540200	0.20467505
131	Matrix Metalloproteinase-10 (MMP-10)	0.091975823	0.15477072	0.20258021
132	Thrombospondin-1	0.092154115	0.15801010	0.20124486
133	Cortisol	0.088743359	0.15172523	0.19191675

134	Thymus-Expressed Chemokine (TECK)	0.10534352	0.16490214	0.18888736
135	Follicle-Stimulating Hormone (FSH)	0.095122948	0.15729684	0.18775593
136	Matrix Metalloproteinase-7 (MMP-7)	0.093772069	0.15445678	0.18691689
137	Placenta Growth Factor (PLGF)	0.095516898	0.15592314	0.18551560
138	Monocyte Chemotactic Protein 1 (MCP-1)	0.097422265	0.15795209	0.18539156
139	Calcitonin	0.084548436	0.14433114	0.18526480
140	Fas Ligand (FasL)	0.085838482	0.14307798	0.17690554
141	Heparin-Binding EGF-Like Growth Factor	0.081296846	0.13595735	0.17655917
142	Macrophage Inflammatory Protein-3 alpha	0.082029037	0.13756849	0.16991971
143	Luteinizing Hormone (LH)	0.079898305	0.13378154	0.16510352
144	Interleukin-13 (IL-13)	0.071049206	0.12123019	0.15323018
145	Betacellulin (BTC)	0.074482180	0.12447471	0.15229923
146	Serum Glutamic Oxaloacetic Transaminase	0.053348403	0.092916518	0.12017415

Proteins were sorted according to their total LOAD-abnormality levels ($Ab^{HC\rightarrow AD}$).

Supplementary Table 3. LOAD-abnormality indices obtained for the CSF proteins.

Order	CSF Proteins	$Ab^{HC\rightarrow EMCI}$	$Ab^{HC\rightarrow LMCI}$	$Ab^{HC\rightarrow LOAD}$
1	Fatty Acid-Binding Protein, heart (FABP)	0.36181390	0.62887013	0.80098552
2	Cortisol	0.31363991	0.53850079	0.67405176
3	Apolipoprotein(a) (Lp(a))	0.30752039	0.53113961	0.67150795
4	Tau	0.22644106	0.40953469	0.56089634
5	Prolactin (PRL)	0.20242180	0.37121323	0.50939101
6	pTau	0.22530790	0.39239028	0.50848424
7	TNF-Related Apoptosis-Inducing Ligand Re	0.21088803	0.37487242	0.49515954
8	Chemokine CC-4 (HCC-4)	0.21000296	0.35266677	0.42933312
9	Macrophage Migration Inhibitory Factor	0.17786901	0.31007376	0.40123689
10	Ferritin (FRTN)	0.19620298	0.32914191	0.39938426
11	Serum Glutamic Oxaloacetic Transaminase	0.19343799	0.32547793	0.39915705
12	Follicle-Stimulating Hormone	0.19624069	0.32761067	0.39590704
13	Amyloid-Beta	0.18330145	0.31130910	0.39309517
14	Sortilin	0.19559284	0.32562995	0.39272994
15	Heparin-Binding EGF-Like Growth Factor	0.20333542	0.32983619	0.38706464
16	Alpha-1-Antitrypsin (AAT)	0.19920903	0.32484692	0.37855604
17	CD 40 antigen (CD40)	0.22155049	0.34434968	0.37799481
18	Resistin	0.20128210	0.32440045	0.37556225
19	Angiotensin-Converting Enzyme (ACE)	0.19154605	0.31556308	0.37464759
20	S100 calcium-binding protein B (S100-B)	0.18148431	0.30188203	0.36276838
21	Alpha-2-Macroglobulin (A2Macro)	0.17552529	0.29564339	0.36179847
22	Monokine Induced by Gamma Interferon (MI	0.17576262	0.29530829	0.35930553
23	Vascular Endothelial Growth Factor (VEGF)	0.15990482	0.27684775	0.35624531
24	Tumor Necrosis Factor Receptor 2 (TNFR2)	0.16166167	0.27821544	0.35291472
25	Cancer Antigen 19-9 (CA-19-9) (U/mL)	0.19813977	0.30902529	0.34935170

26	Sex Hormone-Binding Globulin (SHBG)	0.18162325	0.29689682	0.34799936
27	Macrophage Colony-Stimulating Factor 1	0.17364226	0.28918898	0.34799895
28	Neutrophil Gelatinase-Associated Lipocal	0.14673933	0.26011282	0.34763485
29	Placenta Growth Factor (PLGF)	0.17096451	0.28278387	0.33870724
30	Interleukin-6 receptor (IL-6r)	0.18018143	0.29072544	0.33768457
31	N-terminal prohormone of brain natriuret	0.13878338	0.24627545	0.33716851
32	Matrix Metalloproteinase-3 (MMP-3)	0.20995550	0.31637871	0.33713105
33	Interleukin-25 (IL-25)	0.16069016	0.26853499	0.32562283
34	Hepatocyte Growth Factor (HGF)	0.13342956	0.23688295	0.32171121
35	Pregnancy-Associated Plasma Protein A (P (mIU/mL)	0.14943373	0.25480938	0.31897607
36	Intercellular Adhesion Molecule 1 (ICAM-1)	0.14791262	0.25278771	0.31715426
37	Chromogranin-A (CgA)	0.14605744	0.24967143	0.31626049
38	Apolipoprotein C-III (Apo C-III)	0.15538265	0.25899455	0.31443152
39	C-Reactive Protein (CRP)	0.14064625	0.24203691	0.31166780
40	Macrophage Inflammatory Protein-1 beta	0.15177649	0.25401592	0.30865231
41	Clusterin (CLU)	0.15311813	0.25534129	0.30792642
42	Stem Cell Factor (SCF)	0.15289271	0.25126353	0.30612752
43	Adiponectin	0.14062977	0.23765895	0.30590519
44	Beta-2-Microglobulin (B2M)	0.14471985	0.24471220	0.30259487
45	Alpha-1-Microglobulin (A1Micro)	0.14163253	0.24058451	0.30087751
46	Tissue Inhibitor of Metalloproteinases 1	0.16774495	0.26488861	0.29950941
47	Agouti-Related Protein (AGRP)	0.14900728	0.24682379	0.29806229
48	Apolipoprotein A-I (Apo A-I)	0.14563188	0.24268633	0.29636481
49	AXL Receptor Tyrosine Kinase (AXL)	0.12418669	0.21954800	0.29556876
50	Complement C3 (C3)	0.14556798	0.24320114	0.29484558
51	Fibrinogen	0.14668840	0.24343631	0.29365304
52	Pancreatic Polypeptide (PPP)	0.15994699	0.24809407	0.29351482
53	Immunoglobulin A (IgA)	0.14309230	0.23976347	0.29328507
54	Monocyte Chemotactic Protein 2 (MCP-2)	0.13087383	0.22762759	0.29227412
55	Endothelin-1 (ET-1)	0.13613954	0.22961701	0.29099184
56	Trefoil Factor 3 (TFF3)	0.14643638	0.24246739	0.28950238
57	Myoglobin	0.15921240	0.25350466	0.28777656
58	Leptin	0.16327105	0.25660637	0.28537861
59	Osteopontin	0.13837206	0.23183689	0.28470275
60	Tissue Factor (TF)	0.14721081	0.24031155	0.28344741
61	Apolipoprotein H (Apo H)	0.14538109	0.23726633	0.28340924
62	Calcitonin	0.14541917	0.23858829	0.28335780
63	Apolipoprotein E (Apo E)	0.13802728	0.23024778	0.27891430
64	Prostatic Acid Phosphatase (PAP)	0.14327289	0.23382910	0.27629402
65	Interleukin-16 (IL-16)	0.13808469	0.22904900	0.27527452
66	Thyroxine-Binding Globulin (TBG)	0.14627187	0.23229627	0.27287713
67	Thrombomodulin (TM)	0.12897258	0.21738179	0.26660836
68	Lectin-Like Oxidized LDL Receptor 1	0.13998823	0.22495426	0.26116693
69	Cystatin-C	0.13331831	0.21946424	0.26078132
70	Plasminogen Activator Inhibitor 1 (PAI-1)	0.12314712	0.20852247	0.26013628
71	Fibroblast Growth Factor 4 (FGF-4)	0.12281058	0.20821384	0.25981063

72	Angiopoietin-2 (ANG-2)	0.12552603	0.21036042	0.25714210
73	Interferon gamma Induced Protein 10	0.12027795	0.20198762	0.25080526
74	Vascular Cell Adhesion Molecule-1 (VCAM-1)	0.11843129	0.20047462	0.24964041
75	Interleukin-3 (IL-3)	0.11608233	0.19786201	0.24867901
76	von Willebrand Factor (vWF)	0.12270322	0.20212851	0.24129108
77	Transforming Growth Factor alpha (TGF-al)	0.11685932	0.19436575	0.23432750
78	Serum Amyloid P-Component (SAP)	0.10844245	0.18485488	0.23317331
79	Matrix Metalloproteinase-2 (MMP-2)	0.11644477	0.19188574	0.23308001
80	T Lymphocyte-Secreted Protein I-309	0.11616259	0.19147493	0.23077227
81	Interleukin-8 (IL-8)	0.10877649	0.18366803	0.22925989
82	Insulin-like Growth Factor-Binding Prote	0.10969035	0.18574958	0.22925879
83	Apolipoprotein D (Apo D)	0.11681202	0.18917660	0.22687773
84	Monocyte Chemotactic Protein 1 (MCP-1)	0.10939970	0.18166694	0.22092666
85	Fas Ligand (FasL)	0.11677453	0.18668705	0.21848528
86	Alpha-Synuclein	0.077585667	0.14296047	0.19708815
87	T-Cell-Specific Protein RANTES (RANTES)	0.074124731	0.12754099	0.16586235

Proteins were sorted according to their total LOAD-abnormality levels ($Ab^{HC\rightarrow AD}$).

Supplementary Note 1. Algorithmic details for model evaluation.

Algorithm:

1. Cognitive "homogenization" of clinical groups:
    1.1. For all clinical groups, removal of subjects that presented clinical conversions.
    1.2. For each clinical group, removal of subjects that presented a low likelihood (under 10th percentile) of belonging (cognitively) to the given group. Individual likelihoods were calculated using as reference a multivariate normal distribution defined by the data from the corresponding clinical group. Cognitive evaluations were based on five specific scores known to be sensitive to LOAD progression [2]: the Mini Mental State Examination (MMSE), the Alzheimer's Disease Assessment Scale-cognitive subscales (ADAS11 and ADAS13), the Clinical Dementia Rating Sum of Boxes (CDRSB), and the Rey Auditory Verbal Learning Test (RAVLT30).
2. For each biomarker *i* (i=1..N_{bios}), with subjects indexed by *j* (j=1..N_i):
    2.1. Outlier detection, with a significant squared Mahalanobis distance (P < 0.05) meaning an outlier [3]. For spatial (neuroimaging) biomarkers, the detection of ourliers was performed only once, based on the multivariate Mahalanobis distances obtained for the corresponding imaging modality, with brain regions defining the multivariate space.

2.2. Bootstrapping replication *r* (*r=1..N*$_{boots}$; here N_{boots}=500):

2.2.1. Creation of a different data set, considering replacements across subjects. The selected subjects keep their original longitudinal points.

2.2.2. For each created data set, standardizing the biomarker levels (i.e. centering to have mean 0 and scaling to have standard deviation 1).

2.2.3. Models fitting (Equation 3) and model selection (i.e. using the *Bayesian Information Criterion* to select between the purely fixed effects model, the mixed effects model with different intercepts and fixed slopes, or the mixed effects model with different time intercepts and different slopes).

2.2.4. For the fitted fixed effects parameters (Equation 3), averaging the obtained analytic expression by gender, educational level and apoe-e4, from their minimum to their maximum values:

$$\hat{y}_i(age, DS) = \frac{1}{C}\iiint_{gender_{min}, edu_{min}, apoe\mathcal{E}4_{min}}^{gender_{max}, edu_{max}, apoe\mathcal{E}4_{max}} \widehat{f_i}(age, DS, gender, edu, apoe\mathcal{E}4)\,\mathrm{d}gender\,\mathrm{d}edu\,\mathrm{d}apoe\mathcal{E}4$$

$$C = \frac{1}{(gender_{max} - gender_{min})(edu_{max} - edu_{min})(apoe\mathcal{E}4_{max} - apoe\mathcal{E}4_{min})}.$$

Note that as gender and apoe-e4 are categorical variables, the integration over these factors is equivalent to the sum over all their possible values.

2.2.5. Generation of multiple characteristic trajectories for each possible aging and clinical transition. For each transition, age and *DS* changes linearly (or following a sigmoid function; see Supplementary Fig. 1) from their minimum/baseline (age = 40, and DS = 1) to their maximum values (age = 70, and DS = 1, 2, 3 or 4 for a HC to HC, HC to EMCI, HC to LMCI or HC to LOAD transitions, respectively). Each trajectory consists of multiple time points (here 1560 increasing values, i.e. one point for every week).

2.2.6. Calculation of the temporal ($D_i^{HC\rightarrow LOAD}(age, DS)$) and total ($Ab_i^{HC\rightarrow LOAD}$, $Ab_i^{HC\rightarrow EMCI}$, $Ab_i^{HC\rightarrow LMCI}$) abnormality indexes, using expressions (4) and (5) respectively.

2.3. Calculation of mean and 95 % confidence intervals (*CI)* for each aging/disease trajectory and their associated abnormality indexes, based on all the bootstrap outcomes.

3. For spatial (neuroimaging) biomarkers, creation of an average abnormality trajectory across brain regions, weighting each region according to its total (multi-factorial) abnormality level (see Figs. 3b and 4).

References

1. Jack, C. R. *et al.* Tracking pathophysiological processes in Alzheimer's disease: an updated hypothetical model of dynamic biomarkers. *Lancet Neurol.* **12,** 207–16 (2013).
2. Jedynak, B. M. *et al.* A computational neurodegenerative disease progression score: Method and results with the Alzheimer's disease neuroimaging initiative cohort. *Neuroimage* (2012). doi:10.1016/j.neuroimage.2012.07.059
3. Trujillo-Ortiz, A., Hernandez-Walls, R., Castro-Perez, A. & Barba-Rojo., K. MOUTLIER1:Detection of Outlier in Multivariate Samples Test. *A MATLAB file [WWW Doc.* (2006). at <http://www.mathworks.com/matlabcentral/fileexchange/loadFile.do?objectId=12252>
